# On the subjective acceptance during cardiovascular magnetic resonance imaging at 7.0 Tesla

**DOI:** 10.1186/1532-429X-17-S1-P13

**Published:** 2015-02-03

**Authors:** Sabrina Klix, Antje Els, Katharina Paul, Andreas Graessl, Celal Oezerdem, Oliver Weinberger, Lukas Winter, Christof Thalhammer, Till Huelnhagen, Jan Rieger, Heidrun Mehling, Jeanette Schulz-Menger, Thoralf Niendorf

**Affiliations:** 1 Berlin Ultra-High Field Facility, Max Delbrück Center for Molecular Medicine, Berlin, Germany; 2 Experimental and Clinical Research Center (ECRC), a joint cooperation between the Charité Medical Faculty and the Max-Delbrueck-Center for Molecular Medicine, Berlin, Germany

## Background

A growing number of reports speak about explorations into cardiovascular magnetic resonance (CMR) at ultrahigh magnetic field strengths (UHF-CMR, B_0_≥7.0T). *En route* to broader UHF-CMR studies it is of relevance to scrutinize how UHF-CMR examinations are tolerated by subjects. Realizing this need this study examines the subjective acceptance during UHF-CMR in a cohort of healthy volunteers who underwent a cardiac MR examination at 7.0 T.

## Methods

Within a period of two and a half years (January 2012 to June 2014) a total of 165 healthy volunteers (41 female, 124 male) without any known history of cardiac disease underwent UHF-CMR. For the assessment of the subjective acceptance a questionnaire was used to examine the participants experience prior, during and after the UHF-CMR examination. For this purpose, the subjects were asked to respond to the questionnaire in an exit interview held immediately after the completion of the UHF-CMR examination under supervision of a study nurse to ensure accurate understanding of the questions. All questions were answered with "yes" or "no" including extra space for additional comments.

## Results

Transient muscular contraction was documented in 12.7% of the questionnaires. Muscular contraction was reported to occur only during periods of scanning with the magnetic field gradients being rapidly switched. Dizziness during the study was reported by 12.7% of the subjects. Taste of metal was reported by 10.1% of the study population. Light flashes were reported by 3.6% of the entire cohort. 13% of the subjects reported side effects/observations which were not explicitly listed in the questionnaire but covered by the question about other side effects and observations. No severe side effects as vomiting or syncope after scanning occurred. No increase in heart rate was observed during the UHF-CMR exam versus the baseline clinical examination.

## Conclusions

This study adds to the literature by detailing the subjective acceptance of cardiovascular magnetic resonance imaging examinations at a magnetic field strength of 7.0 T. Cardiac MR examinations at 7.0 T are well tolerated by healthy subjects. Broader observational and multi-center studies including patient cohorts with cardiac diseases are required to gain further insights into the subjective acceptance of UHF-CMR examinations.

## Funding

N/A.

**Figure 1:**
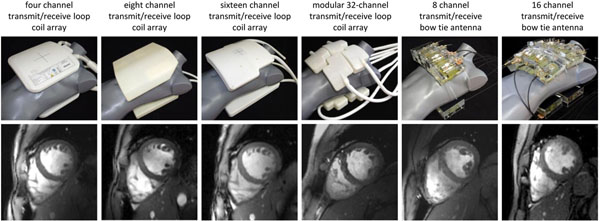
**Synopsis of RF coil configurations used in this study.Top:** Picture photographs of the cardiac optimized 7.0 T transceiver RF coil arrays to illustrate the coil design and the coil geometry together with the coil positioning used in the UHF-CMR setting. The RF coil employed include a four channel [1], an eight channel [2], a 16 channel [3,4] and a 32 channel loop coil [5] configuration and an eight channel [6] and 16 channel bow tie antenna array configuration [7]. **Bottom:** Short axis views of the heart derived from 2D CINE FLASH acquisitions using the RF coil configurations in the top row and a spatial resolution of (1.4 x 1.4 x 4) mm^3^ and parallel imaging (R=2, GRAPPA reconstruction).

**Figure 2:**
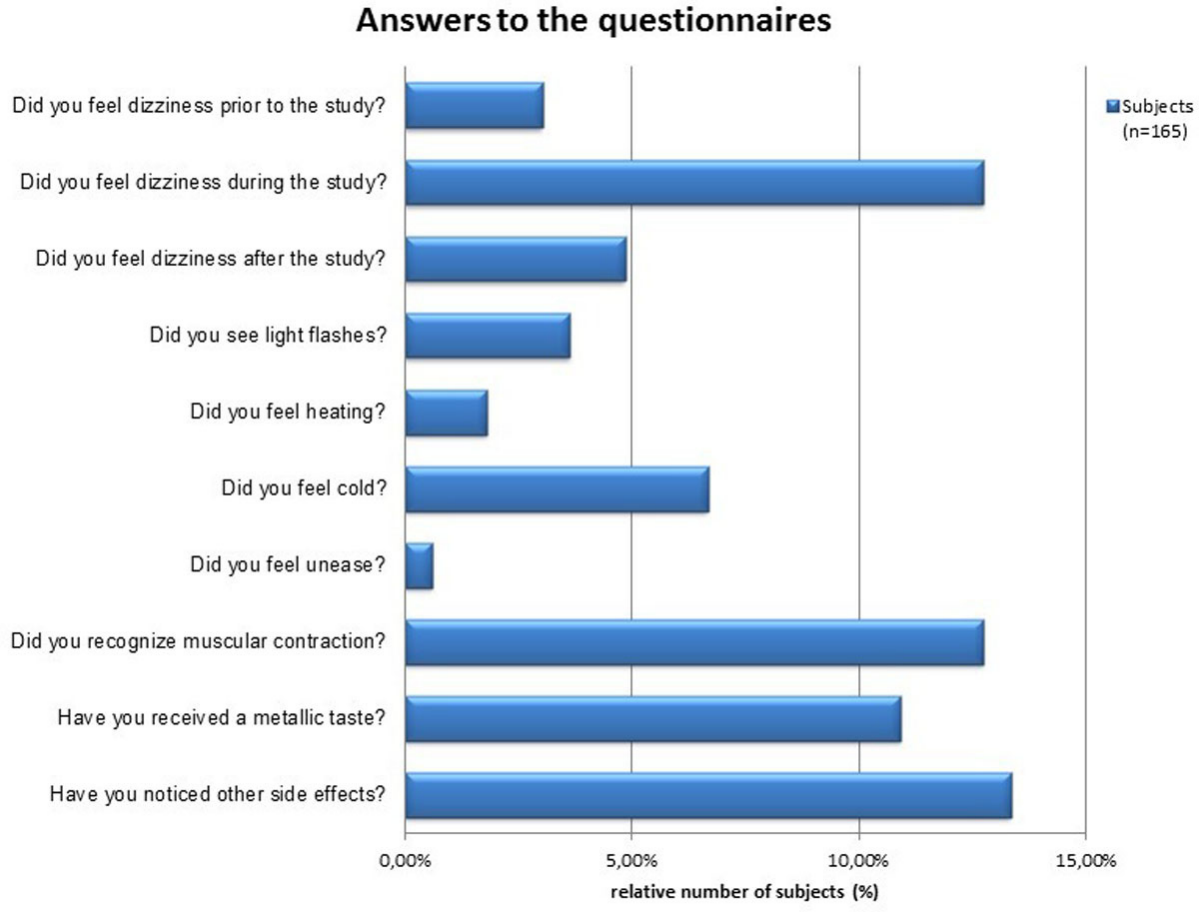
**Results derived from the completed questionnaires.** Synopsis of the results reported by 165 subjects on subjective acceptance of UHF-CMR. The most mentioned side effects reported were transient muscular contraction during scanning (12.7%) and dizziness experienced during the study (12.7%).
